# P-1892. Morning Report 2.0: Unleashing AI for Interactive Progressive Case Discussions

**DOI:** 10.1093/ofid/ofaf695.2061

**Published:** 2026-01-11

**Authors:** Elizabeth W Covington, Amber M Martin, Courtney S Watts Alexander

**Affiliations:** Auburn University Harrison College of Pharmacy, AUBURN, AL; Auburn University Harrison College of Pharmacy, AUBURN, AL; Auburn University Harrison College of Pharmacy, AUBURN, AL

## Abstract

**Background:**

A progressive disclosure case activity utilizing generative artificial intelligence (AI) was implemented within an experiential pharmacy course. Modeled after “morning report,” the activity utilized a generative AI prompt to generate sequential infectious disease patient case details upon prompting from the end user. Students engaged in a weekly interactive discussion to collaboratively complete the activity with student peers and a preceptor. This study aimed to assess the impact of the activity on pharmacy student utilization and perceptions of AI and critical thinking.

**Figure:**
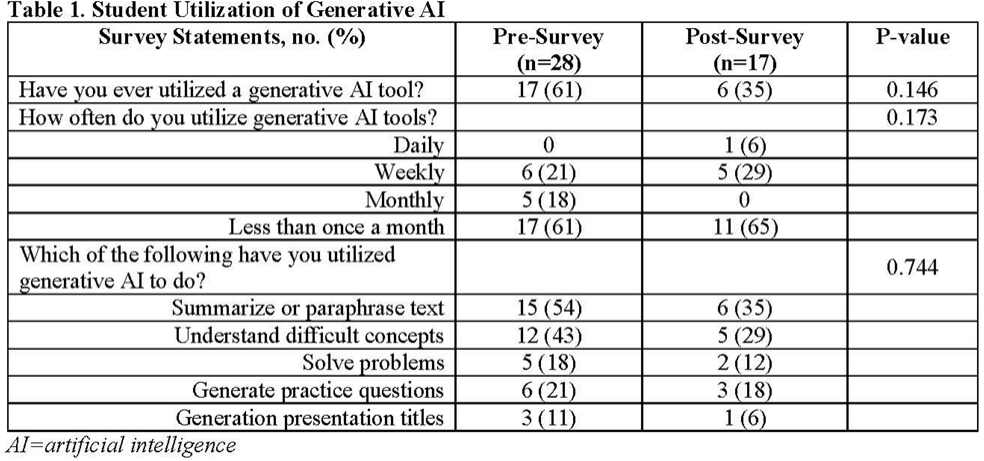

**Figure:**
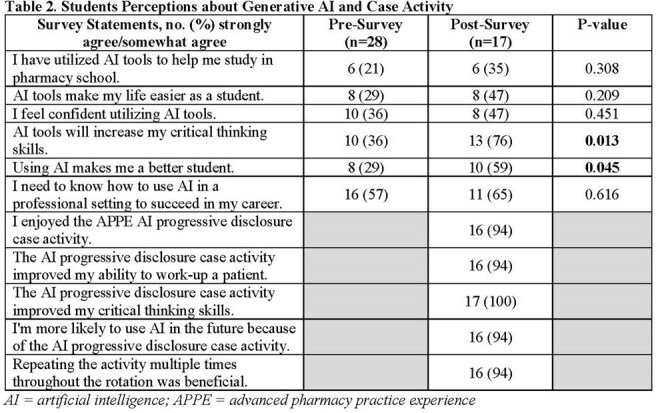

**Methods:**

An embedded mixed-methods pilot study was performed. Pre and post Qualtrics surveys measured quantitative and qualitative student feedback. Nominal data were assessed via Chi square or Fisher’s exact test using SPSS. Correlations were assessed via Pearson’s coefficient. Qualitative free-text survey responses were evaluated by thematic analysis. The study was deemed exempt by the institutional review board at Auburn University.

**Figure:**
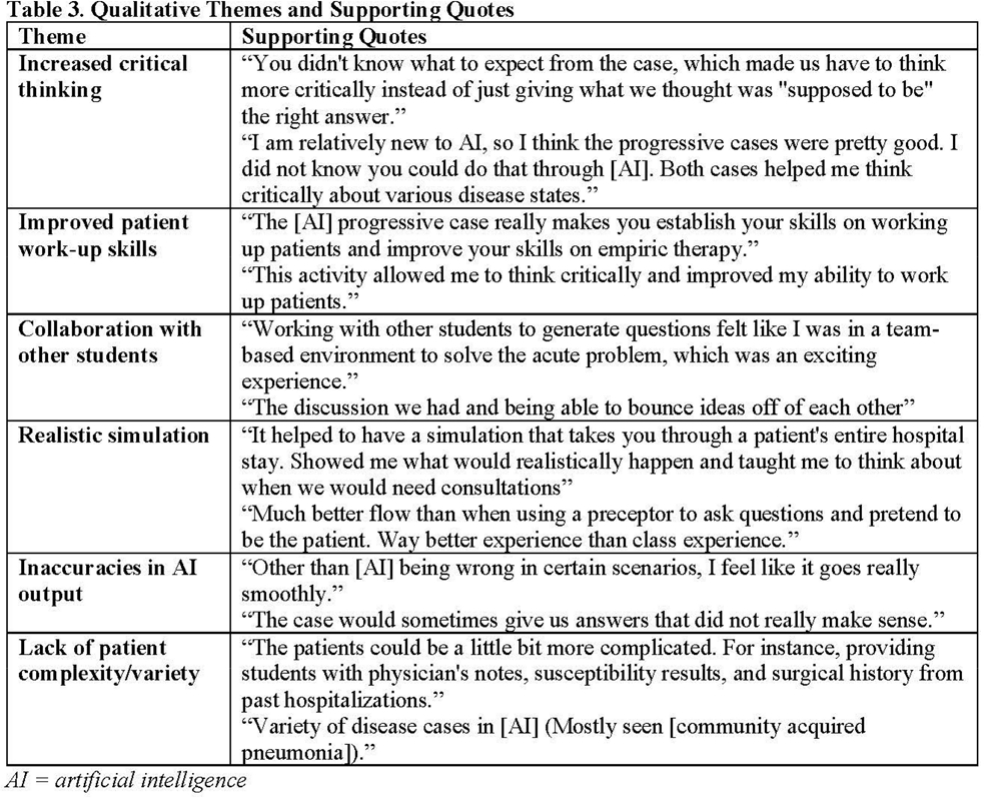

**Results:**

A total of 28 students completed the activity and the pre-survey, while 17 students completed the post-survey (61% response rate). Following activity participation, more students agreed/strongly agreed that AI increases critical thinking skills (36% pre vs. 76% post; P=0.013) and that AI makes them a better student (29% pre vs. 59% post; P=0.045). Older age was negatively correlated with belief in AI improving critical thinking skills (-0.488, P=0.008). The post-survey showed 94% of participants enjoyed the activity, and all agreed it improved their critical thinking. Thematic analysis indicated that the activity promoted critical thinking, patient work-up skills, and collaboration, while providing a realistic case activity. Students also commented on occasional inaccuracies in AI output and limited variety of disease cases.

**Conclusion:**

A generative AI progressive disclosure case activity increased student perceptions of critical thinking and patient work-up skills. These findings highlight the potential for generative AI to facilitate critical thinking skills among pharmacy students. Future iterations of the activity will explore training a custom generative pre-trained transformer and increased case variety.

**Disclosures:**

All Authors: No reported disclosures

